# Neutrophil-to-Lymphocyte, Monocyte-to-Lymphocyte and Platelet-to-Lymphocyte Ratios in Relation to Clinical Parameters and Smoking Status in Patients with Graves’ Orbitopathy—Novel Insight into Old Tests

**DOI:** 10.3390/jcm9103111

**Published:** 2020-09-26

**Authors:** Joanna Szydełko, Michał Litwińczuk, Magdalena Szydełko, Beata Matyjaszek-Matuszek

**Affiliations:** 1 Department of Endocrinology, Medical University of Lublin, Jaczewskiego 8, 20-954 Lublin, Poland; bmm@2com.pl; 2 Department of Endocrinology, Independent Public Clinical Hospital No. 4 in Lublin, Jaczewskiego 8, 20-954 Lublin, Poland; mlitwinczuk405@gmail.com (M.L.); mszydelko@interia.pl (M.S.)

**Keywords:** neutrophil-to-lymphocyte ratio, monocyte-to-lymphocyte ratio, platelet-to-lymphocyte ratio, Graves’ orbitopathy, smoking

## Abstract

Graves’ orbitopathy (GO) is an autoimmune disease with a chronic inflammatory background. Smoking behavior is the main environmental factor responsible for the transition of this major extra thyroidal manifestation of Graves’ disease (GD) from the subclinical to the overt form. Complete blood count-derived parameters are suggested to be novel inflammatory indices. The aim of this retrospective study was to investigate the association between neutrophil-to-lymphocyte (NLR), monocyte-to-lymphocyte (MLR), and platelet-to-lymphocyte ratios (PLR) with selected clinical parameters and smoking status in 406 GD patients with (*n* = 168) and without GO (*n* = 238). The control group consisted of 100 healthy individuals. The activity of GO was graded according to Clinical Activity Score. Significantly higher white blood cells (WBC), neutrophil, and NLR (*p* < 0.05) values were observed in GD patients with GO compared with those without GO. PLR values were significantly higher in GO patients than in the controls. WBC (6.81 ± 1.56 vs. 5.70 ± 1.23) and neutrophils (3.89 ± 1.06 vs. 3.15 ± 0.95) count was higher in active GO patients than in those with inactive GO. Positive correlation (*p* < 0.05) between CAS score and WBC, neutrophil and monocyte count, and NLR was found. Smoking was associated with higher WBC (*p* = 0.040), neutrophil (*p* = 0.049), PLR (*p* = 0.032) values. Multivariate analysis revealed that WBC, NLR may be risk factors for GO development. WBC, neutrophil, NLR and PLR values seem to be useful tools in the assessment of inflammation in GD.

## 1. Introduction

Graves’ orbitopathy (GO) constitutes the major extra thyroidal manifestation of Graves’ disease (GD). GO is an autoimmune inflammatory disorder affecting orbital soft tissue, which may lead to the visual disturbances due to extraocular muscle involvement, proptosis, conjunctival inflammation and oedema [1,2]. Generally, GO occurs in case of Graves’ hyperthyroidism (90%), although it may also be seen in euthyroid (7%) or hypothyroid (3%) patients as a result of chronic autoimmune (Hashimoto) thyroiditis [3]. The clinical course of GO is commonly mild to moderate, and the incidence of severe sight-threatening form ranges from 3 to 5%. It is well established that about 25–35% GD hyperthyroid patients develop clinically manifest GO, although the recent studies revealed that subclinical eye involvement is observed in radiological examinations in nearly 65–75% individuals [3,4]. Thus, there is a great need to investigate early biomarkers to identify GD patients with high risk of GO development.

B-cell lymphocyte-mediated and T lymphocyte-dependent interactions, leading to inflammation and autoimmune processes with the production of antibody against thyroid-stimulating hormone receptor (TRAb), are concerned to play a crucial role in the pathogenesis of GO along with a combination of genetic and environmental factors [5]. Among them, the consistent association between smoking and the occurrence of GO, its severity and progression is widely discussed [6,7,8,9]. Smoking cigarettes affects both the innate and adaptive immune systems. It might exaggerate systemic and local inflammation by releasing pro-inflammatory cytokines and inhibiting anti-inflammatory mediators [10,11]. However, the exact mechanism by which tobacco affects the thyroid function and increases the risk of GO remains unclear.

As cost-effective, easily-accessible and commonly used indices complete blood count (CBC)-derived parameters have been a subject of high interest among researchers over the past decade. Neutrophils are treated as the active components of inflammation, whereas lymphocytes are involved in regulatory and protective pathways [12]. Several studies have proved neutrophil-to-lymphocyte ratio (NLR), monocyte-to-lymphocyte ratio (MLR), platelet-to-lymphocyte ratio (PLR), and mean platelet volume-to-platelet ratio (MPV/PLT) as novel biomarkers of chronic subclinical inflammation in diabetes mellitus, coronary artery disease, different types of malignancies [13,14,15,16]. Moreover, NLR and PLR could serve as indicators to differentiate malignant from benign thyroid nodules in preoperative period as well as they may be predictors of response to treatment, progression of the disease, risk of recurrence, and mortality rate in thyroid cancers [17,18,19,20]. Literature data about the role of NLR, MLR and PLR in chronic autoimmune thyroid diseases are still lacking [21,22,23,24,25,26,27]. There are only single studies evaluating the relationship between these direct and indirect blood-cell associated inflammatory markers and GO [28,29].

Therefore, the aim of the present study was to investigate the association between white blood cells (WBC) parameters, NLR, MLR, PLR, MPV/PLT and the clinical phenotype in GD patients with and without GO, particularly with respect to the smoking status.

## 2. Materials and Methods

### 2.1. Statement of Ethics

The study protocol was approved on 27 February, 2020 by the Local Bioethical Committee of the Medical University of Lublin, Poland (approval No. KE-0254/44/2020). Due to the retrospective nature of the study, informed consent was not required and patients’ data were used anonymously. The research was conducted in accordance with Good Clinical Practice (Declaration of Helsinki of 1975).

### 2.2. Study Design and Patients

**Figure 1:**
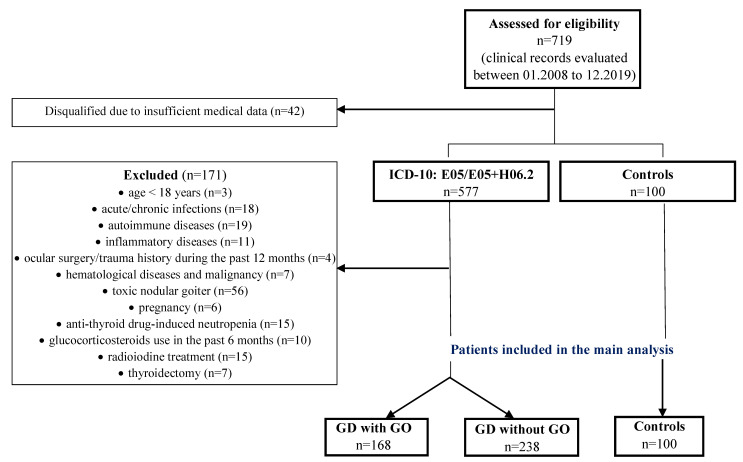
Flow chart of the study.

The research was designed as a retrospective, cross-sectional, and observational, single-centre study. Patients’ data were obtained from paper-based and/or electronic charts. The medical records of 619 patients identified using codes E05.0 and H06.2 according to the International Classification of Diseases revision 10 (ICD-10), hospitalized in the Department of Endocrinology Medical University of Lublin (Poland) during the period between 1 January 2008 and 31 December 2019 were reviewed. All patients diagnosed with GD with and without GO were initially included. The diagnosis of GD was based on the presence of conventional symptoms of hyperthyroidism and confirmed by the laboratory results involving positive serum thyroid-stimulating hormone receptor antibody (TRAb, positive titer > 1.75 IU/L), elevated levels of free triiodothyronine (fT3), free thyroxine (fT4), and decreased levels of thyroid-stimulating hormone (TSH). GO was defined according to the European Group on Graves’ Orbitopathy (EUGOGO) consensus [30]. The control group consisted of 100 randomly selected, healthy individuals with normal thyroid function. The exclusion criteria for the study and control groups were as follows: age < 18 years, acute or chronic infection, other autoimmune/inflammatory systemic or ocular diseases, ocular surgery or trauma history during the past 12 months and permanent traumatic eye injury at any time in the past, the use of ocular or systemic drugs with the proven effect on blood counts, except for thyrostatic drugs used in the therapy of hyperthyroidism in the course of GD (only patients with anti-thyroid drug-induced neutropenia with neutrophil count < 1.5 × 10^9^/L were excluded), glucocorticosteroids used in the past 6 months, pregnancy, past or present malignancy history and hematological disorders, thyroidectomy, radioiodine treatment prior or after the diagnosis of GD, and toxic nodular goiter [31]. Moreover, patients with incomplete medical documentation were also excluded from the study. After evaluation for eligibility, 406 GD patients with and without GO (*n* = 168 vs. *n* = 238, respectively) were enrolled in the analysis. The inclusion and exclusion criteria, and inclusion chart are presented in Figure 1.

Sociodemographic data, such as age, sex, body mass index (BMI) calculating using typical formula: weight (in kilograms) divided by height (in meters) squared, and smoking status were collected. All patients were categorized as never smokers, current smokers or former smokers. A current smoker was defined as an individual who has smoked >100 cigarettes (including hand-rolled cigarettes, cigars and cigarillos) throughout their life and has smoked in the last 28 days, former smoker as someone who has smoked >100 cigarettes in their lifetime but has not smoked in the last 28 days, and never smoker as a person who has not smoked ≤100 cigarettes throughout their life and does not currently smoke. Occasional smokers were treated as current smokers, assuming they have smoked >100 cigarettes in their lifetime [32,33]. Taking into account the greater impact of acute than chronic use of tobacco on CBC-derived inflammatory markers, all GD patients and the controls were next classified into two groups as smokers and non-smokers, the latter including both never smokers and former smokers [34]. Furthermore, duration of smoking and the number of cigarettes smoked per day were determined.

### 2.3. Clinical and Laboratory Assessments

The clinical course of GD and/or GO, including thyroid function and duration of the disease, was characterized. Activity of GO was graded according to the Clinical Activity Score (CAS) involving seven signs, such as spontaneous retrobulbar pain, ocular pain on attempted upward or downward gaze, redness of the eyelids, redness of the conjunctiva, swelling of the eyelids, chemosis of the conjunctiva, inflammation of caruncule and/or plica. One point was given for the presence of each of the parameters and active GO was defined as CAS ≥ 3/7 [2,35]. Additionally, the incidence of subjective symptoms, such as excessive tearing or dry eyes, diplopia, and photophobia were assessed.

TSH, fT3, fT4 concentrations were assessed to determine the thyroid status in both study and control groups. According to the laboratory results, individuals were classified into two groups: euthyroidism with the values of TSH, fT3, fT4 within normal ranges and impaired thyroid function. Thyroid axis dysfunction involved primary (elevated fT3, fT4 concentrations and decreased TSH level) or subclinical hyperthyroidism (decreased TSH level and fT3, fT4 concentrations within normal ranges) in not yet treated or undertreated subjects, and primary (elevated TSH level and decreased fT3, fT4 concentrations) or subclinical hypothyroidism (elevated TSH level and fT3, fT4 concentrations within normal ranges) as a result of overtreatment with anti-thyroid drugs. Moreover, in the studied group, TRAb concentrations were measured.

The CBC data obtained from venous blood samples of all participants from the study and control groups and determined by automated standard laboratory methods were analyzed. The parameters of WBC system and its subpopulations: neutrophil, lymphocyte, monocyte, basophil, eosinophil counts as well as platelet (PLT) counts and mean platelet volume (MPV) were measured. NLR, MLR, PLR values were calculated by dividing the absolute neutrophil, monocyte, and platelet counts by the absolute lymphocyte count, respectively. MPV/PLT ratio was evaluated in all subjects from the controls and in 107 GD with GO patients and 141 those without GO.

### 2.4. Statistical Analysis

Parametric tests were used for the statistical analysis due to the distribution of the examined variables close to normal and the large sample sizes (*n* > 100). Continuous variables are presented as mean ± standard deviation (SD) for each group, and categorical ones are expressed as numbers with percentages (%). Chi-Squared test was performed to determine sex distribution between groups. Student’s *T*-test was used to compare differences between two groups, whereas one-way analysis of variance (ANOVA) test was used for more than 2 groups with Tukey’s post hoc test for unequal n. Assessment of the associations with more than one quantitative variables was performed using two-way ANOVA and Scheffe post-hoc for multiple pair-wise comparisons. The Pearson’s correlation analysis was used to measure the degree of the relationship between linearly related variables. The analysis of correlation in case of CAS scale, due to the numerous repeats of the same values, was performed by the Kendall’s Tau test analysis. Univariate and multivariate logistic regression modelling was performed to identify variables that were significantly and independently associated with the occurrence of GO and were presented with odds ratio (ORs) with 95% confidence intervals (CIs). Multivariate model was estimated using stepwise backward validation and it contained only adjusted ORs for significant parameters. All *p*-values are two-tailed, and *p*-values < 0.05 were considered as statistically significant. The statistical analysis was performed with STATISTICA 13.3.0 software for Windows (TIBCO Software Inc., Palo Alto, CA, USA).

## 3. Results

### 3.1. Baseline Characteristics of Study and Control Group

Mean ages of GD and control groups were 48.3 ± 15.2 and 42.5 ± 17.3 years, respectively. The majority (*n* = 331; 81.5%) of patients in the study group were females. Baseline demographic characteristics of GD patients with and without GO and controls are shown in Table 1.

The mean age, BMI and gender distribution were similar in both GD with and without GO groups, while significant differences were detected with respect to age and gender between two studied groups and controls (*p* < 0.001 vs. *p* = 0.002). Mean duration of GD in the whole study group was 42.1 ± 70.2 months (range: 0–468), while the mean duration of GO was 20.7 ± 47.6 (range 0–356) months.

Regarding hematological parameters, WBC, neutrophil counts and NLR, PLR (for all *p* < 0.001), MLR (*p* = 0.012), and MPV (*p* = 0.028) values were significantly higher in GD patients compared to healthy individuals. The statistically lower value was observed only in case of lymphocyte counts (*p* < 0.001) between the study and control groups (Table S1). The comparisons of hematological, inflammatory parameters and above-mentioned ratios among GD with GO (Group 1), GD without GO (Group 2) subjects and controls (Group 3) are presented in Table 2.

Analysis using one-way ANOVA revealed the significant intergroup differences in mean values of WBC, neutrophil, lymphocyte, NLR, MLR, and PLR. MPV values turned out to be marginally statistically significant (*p* = 0.054) between the groups. The Tukey’s post hoc analysis showed that NLR values were statistically different between all three groups (1 vs. 2 *p* = 0.018; 1 vs. 3 *p* < 0.001; 2 vs. 3 *p* < 0.001). The highest mean NLR value was observed in Group 1 (1.95 ± 0.77), Group 2 (1.76 ± 0.62), and the lowest in Group 3 (1.41 ± 0.21). Moreover, the statistically significant differences between Group 1 and 2 in case of WBC (6.64 ± 2.09 vs. 5.96 ± 1.56; *p* = 0.022) and neutrophil (3.78 ± 1.42 vs. 3.24 ± 1.06; *p* < 0.001) counts were observed.

### 3.2. Differences in Hematological Indices between Active and Inactive Graves’ Orbitopathy

Active GO was found in 143 (85.1%) patients and inactive in only 25 (14.9%) individuals. The most frequently observed signs were redness of the conjunctiva (*n* = 137; 81.5%), chemosis of the conjunctiva (*n* = 130; 77.4%), ocular pain on attempted upward or downward gaze (*n* = 113; 67.3%), swelling of the eyelids (*n* = 93; 55.4%), and spontaneous retrobulbar pain (*n* = 91; 54.2%), whereas redness of the eyelids (*n* = 43; 25.6%) was the least frequently reported one. Only one patient had inflammation of caruncule and plica. Among 168 patients, subjective symptoms, such as excessive tearing or dry eyes (*n* = 125) and diplopia (*n* = 86) were noted in more than half of them. Photophobia was registered in 23 (13.7%) patients. There was significantly higher WBC (6.81 ± 1.56 vs. 5.70 ± 1.23), neutrophil (3.89 ± 1.06 vs. 3.15 ± 0.95) counts, and lower MPV/PLT values (0.03 ± 0.01 vs. 0.04 ± 0.01) in active GO patients than in inactive group (*p* = 0.002, *p* = 0.016, *p* = 0.021, respectively). No statistically significant differences were noted in other assessed hematological parameters and calculated ratios (Figure S1A–L).

**Figure 2:**
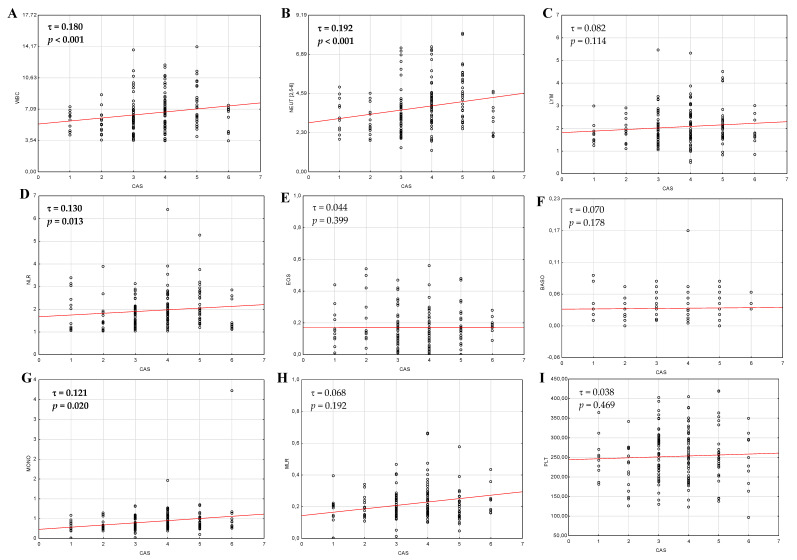

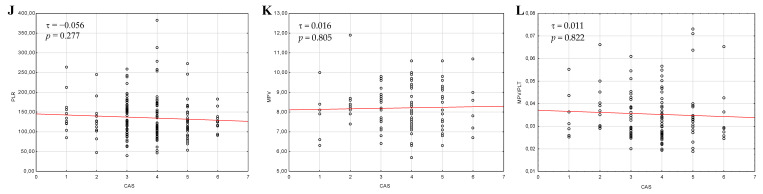
Correlations (Kendall’s tau) of scores in CAS scale and hematological parameters in GO patients. (**A**,**B**,**D**,**G**) The statistically significant positive correlation was observed between WBC, NEU, MONO, NLR and CAS scale. (**C**,**E**,**F**,**H**−**L**) There was no statistically significant correlation between LYM, EOS, BASO, MLR, PLT, PLR, MPV, MPV/PLT and the activity of GO in CAS scale. *p*-value < 0.05 was statistically significant. GO—Graves’ orbitopathy; CAS—Clinical Activity Score; WBC—white blood cell; NEU—neutrophil; LYM—lymphocyte, NLR—neutrophil-to-lymphocyte ratio; EOS—eosinophil; BASO—basophil; MONO—monocyte; MLR—monocyte-to-lymphocyte ratio; PLT—platelet; PLR—platelet-to-lymphocyte ratio; MPV—mean platelet volume.

The differences between CBC parameters and blood-cell derived ratios in relation to particular signs from CAS scale and three subjective symptoms are shown in Table 3. The Kendall’s Tau test analysis revealed statistically important positive monotonic correlations between scores in CAS scale, reflected by the number of points from 1 to 7, and examined parameters (WBC, neutrophil, monocyte, NLR) which are presented in Figure 2. The strength of the monotonic relationship reflected by the Kendall correlation coefficient was τ = 0.180 for WBC, τ = 0.192 for neutrophil, τ = 0.121 for monocyte counts, and τ = 0.130 for NLR values. The statistically significant positive correlation was also investigated between the duration of GO and MPV values (r = 0.194, *p* = 0.045), and significant negative correlation was found in case of PLT counts (r = −0.231, *p* = 0.003) and PLR (r = −0.158, *p* = 0.041) in GO patients. In turn, the duration of GD in GO group was negatively correlated with WBC (r = −0.186, *p* = 0.016), neutrophil (r = −0.188, *p* = 0.015), and PLT (r = −0.176, *p* = 0.023) counts. Among GD patients without GO, none of correlations was noted.

### 3.3. Differences in Hematological Parameters According to Thyroid Status

To analyze hematological parameters and derived ratios in relation to thyroid status, GD patients were categorized according to thyroid test results into two groups: euthyroid (*n* = 72) and subjects with impaired thyroid function (*n* = 334). Patients with thyroid axis dysfunctions had significantly higher neutrophil counts and higher NLR values with no statistical significance compared with euthyroid individuals (3.80 ± 1.38 vs. 3.39 ± 1.21; *p* = 0.022 and 1.99 ± 0.80 vs. 1.81 ± 0.66; *p* = 0.070), whereas monocyte counts and MLR values were significantly lower as compared to those with compensated thyroid function (0.38 ± 0.15 vs. 0.46 ± 0.31 and 0.20 ± 0.07 vs. 0.25 ± 0.22, all *p* < 0.001). TRAb concentration was found to be statistically higher (*p* = 0.048) in euthyroid patients than in those with impaired thyroid function (15.34 ±13.25 vs. 9.37 ± 10.90), probably as a consequence of anti-thyroid treatment.

### 3.4. Differences in Hematological Parameters in the Study and Control Group According to Smoking Status

**Figure 3:**
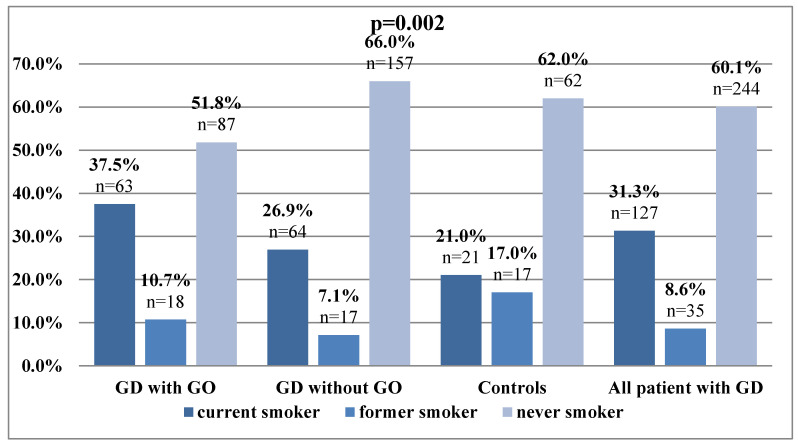
Prevalence of particular smoking status among the study and control groups (*p* = 0.002).

To investigate the impact of tobacco use on CBC-derived inflammatory markers in GD patients with/without GO and the controls, the correlation between smoking status and hematological parameters was performed. Patients with GO reported current tobacco use more frequently (37.5%) than GD patients without GO (26.9%) or healthy subjects (21.0%). The percentage of participants who have never smoked was more than 50% in each of the analyzed groups (Figure 3).

The average time of nicotine addiction and the number of cigarettes smoked per day were significantly higher in GD patients with GO as compared to those without thyroid eye disease (9.4 ± 12.6 vs. 6.1 ± 10.7 cigarettes/day, *p* = 0.003 and 8.1 ± 11.2 vs. 4.4 ± 7.0 years, *p* < 0.001). The Pearson’s correlation analysis displayed the statistically significant negative correlations between the number of cigarettes/day and platelet counts (r = −0.152, *p* = 0.049), PLR values (r = −0.170, *p* = 0.027) in GO group, while the significant positive correlation with MPV (r = 0.212, *p* = 0.012) was noted in GD patients without GO. The analysis revealed that there were significant correlations between the duration of tobacco use and WBC (r = 0.188, *p* = 0.015), neutrophil (r = 0.189, *p* = 0.015), lymphocyte (r = 0.153. *p* = 0.049), and PLR (r = −0.230, *p* = 0.003) in GO patients. Additionally, the positive correlations between WBC (r = 0.156, *p* = 0.016), neutrophil (r = 0.173, *p* = 0.007), and NLR (r = 0.143, *p* = 0.028) and the time of smoking were found in GD without GO group.

**Figure 4:**
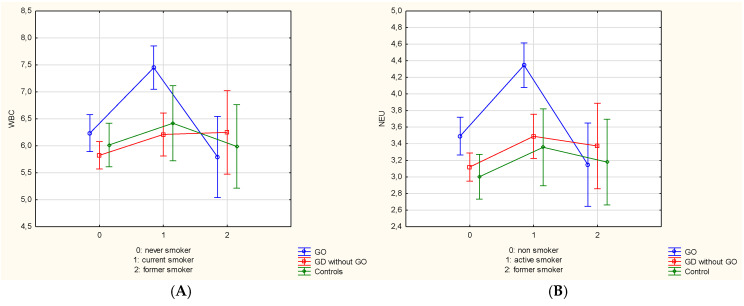
(**A**,**B**) Associations between different smoking statuses and hematological parameters in the study and control population. Vertical bars represent 0.95 confidence intervals. *p*-value < 0.05 was statistically significant. GO—Graves’ orbitopathy; GD—Graves’ disease; WBC—white blood cell; NEU—neutrophil. (**A**) The interaction effect of group and smoking status (*p* = 0.040). GO: current smoker 7.45 ± 2.15, former smoker 5.79 ± 1.58, never smoker 6.23 ± 1.97; GD without GO: current smoker 6.21 ± 1.35, never-smoker 5.82 ± 1.60, former smoker 6.25 ± 1.85; controls: current smoker 6.42 ± 1.02, former smoker 5.99 ± 0.79, never smoker 6.02 ± 0.87. (**B**) The interaction effect of group and smoking status (*p* = 0.049). GO: current smoker 4.35 ± 1.48, former smoker 3.15 ± 1.25, never smoker 3.49 ± 1.27; GD without GO: current smoker 3.49 ± 1.06, never-smoker 3.12 ± 1.05, former smoker 3.37 ± 1.06; controls: current smoker 3.36 ± 0.53, former smoker 3.18 ± 0.47, never smoker 3.00 ± 0.40.

The associations between three smoking statuses and hematological parameters in both studied and control groups are given in Figure 4A,B. The analysis using two-way ANOVA and Scheffe post-hoc test revealed the significant effect of group (*p* = 0.007) and smoking separately (*p* < 0.001) on neutrophil counts, as well as the interaction between smoking and group effects were proved to be statistically significant (*p* = 0.049). No significant group effect was noted in case of WBC counts except for the smoking effect (*p* < 0.001). Moreover, the interaction between group and smoking effects was significant (*p* = 0.040) on WBC level. It proves that smoking affects the WBC count, however it depends on the group. The analysis revealed the significant effect of group (*p* = 0.037) and the interaction between smoking and group effects (*p* = 0.049) on lymphocyte counts. In case of MPV values, the interaction of group and smoking effects showed statistical differences (*p* = 0.040).

**Figure 5:**
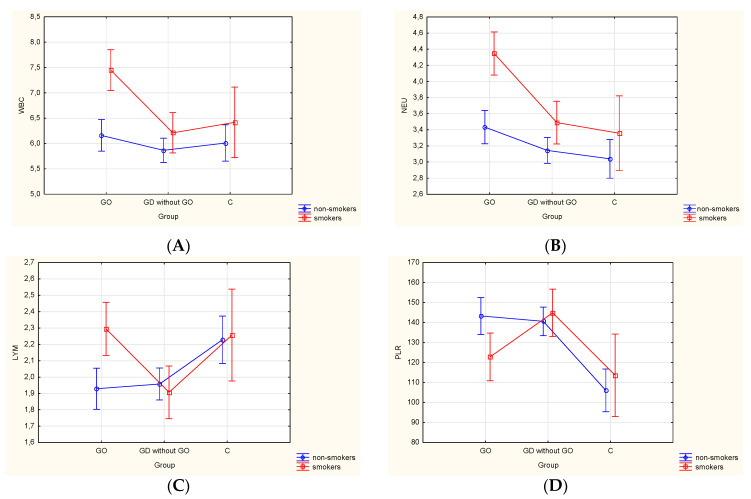
(**A**–**D**) Analysis of the variance resulting from the effect of different factors (smoking status: non-smokers, smokers; presence or absence of GO in GD patients, lack of the disease) on blood count parameters. Vertical bars represent 0.95 confidence intervals. *p*-value < 0.05 was statistically significant. GO—Graves’ orbitopathy; GD—Graves’ disease; WBC—white blood cell; NEU—neutrophil; LYM—lymphocyte; PLR—platelet-to-lymphocyte ratio. (**A**) The interaction effect of group and smoking status (*p* = 0.019). GO: smokers 7.45 ± 2.15, non-smokers 6.16 ± 1.91; GD without GO: smokers 6.21 ± 1.35, non-smokers 5.87 ± 1.63; controls: smokers 6.42 ± 1.02, non-smokers 6.01 ± 0.85. (**B**) The interaction effect of group and smoking status (*p* = 0.033). GO: smokers 4.35 ± 1.48, non-smokers 3.43 ± 1.27; GD without GO: smokers 3.49 ± 1.06, non-smokers 3.14 ± 1.05; controls: smokers 3.36 ± 0.53, non-smokers 3.04 ± 0.42. (**C**) The interaction effect of group and smoking status (*p* = 0.011). GO: smokers 2.29 ± 0.86, non-smokers 1.93 ± 0.71; GD without GO: smokers 1.91 ± 0.58, non-smokers 1.96 ± 0.66; controls: smokers 2.26 ± 0.47, non-smokers 2.23 ± 0.42. (**D**) The interaction effect of group and smoking status (*p* = 0.032). GO: smokers 122.85 ± 47.25, non-smokers 143.30 ± 55.03; GD without GO: smokers 144.87 ± 58.93, non-smokers 140.63 ± 43.51; controls: smokers 112.81 ± 37.78, non-smokers 113.86 ± 29.96.

Next, all GD individuals with and without GO, and the controls were classified into two groups as smokers (*n* = 63; 37.5%/*n* = 64; 26.9%/*n* = 21; 21.0%) and non-smokers (*n* = 105; 62.5%/*n* = 174; 73.1%/*n* = 79; 79%) which included both never-smokers and former smokers. The significant effect of group and smoking separately was observed in WBC and neutrophil counts (all *p* < 0.001) with simultaneous interaction between smoking and group effects (*p* = 0.019 and *p* = 0.033). The analysis displayed the significant effect of group in lymphocyte counts (*p* = 0.002), NLR (*p* = 0.015), MLR (*p* = 0.033), and PLR (*p* < 0.001), whereas additionally the interaction between smoking and group effects were recorded in lymphocyte counts (*p* = 0.011) and PLR values (*p* = 0.032) (Figure 5A–D).

### 3.5. Factors Associated with Higher Risk of Graves’ Orbitopathy Development

To investigate which laboratory data and sociodemographic parameters were most associated with GO occurrence, the logistic regression modeling was performed.

The following four variables showed a significant relationship with GO in the univariate analysis: WBC (OR, 1.232; 95% CI, 1.101–1.380; *p* < 0.001), neutrophil (OR, 1.421; 95% CI, 1.203–1.680; *p* < 0.001) counts, NLR (OR, 1.468; 95% CI, 1.093–1.970; *p* = 0.011), and smoking (OR, 1.656; 95% CI, 1.084–2.531; *p* = 0.020) (Table 4). In the multivariate analysis, greater GO risk was associated with higher WBC counts (OR, 1.209; 95% CI, 1.078–1.355; *p* = 0.001) and NLR values (OR, 1.348; 95% CI, 1.078–1.355; *p* = 0.048).

## 4. Discussion

To the best of our knowledge, this study is the first to comprehensively assess peripheral blood picture and novel inflammatory biomarkers, such as NLR, MLR, PLR, MPV/PLT, and MPV in relation to selected clinical parameters and smoking status in GD patients with and without GO. Recent studies have introduced these simple hematological measurements of immune response as indicators of systemic inflammation in various clinical conditions, from infections by autoimmune diseases to malignancies [15,36]. However, little is known about the above-mentioned markers in thyroid disorders. Several authors proved that NLR may be a useful parameter in the differential diagnosis of benign thyroid nodules from malignant tumors, especially papillary thyroid carcinoma [17,37,38]. Aside from thyroid neoplasms, NLR combined with PLR have been studied as potential indicators of Hashimoto’s disease occurrence, which can allow to distinguish patients with chronic autoimmune thyroiditis from healthy individuals [22,23,24,25].

GD is one of the most common form of hyperthyroidism in iodine-sufficient countries, with the peak of incidence between 40–60 years [35,39]. In our study, the mean age of the disease as well as more common prevalence of GD in women, were similar to the general statistics [40,41]. GO, occurring in about 25–35% of GD, is characterized by production of TRAb against TSH receptor on the surface of orbital fibroblast. In addition to this, the inflammatory background of GO is widely discussed [3,27,42,43]. Our findings demonstrated that both neutrophil counts, and NLR values were significantly elevated in GO patients compared with those without GO or healthy individuals. Furthermore, there were statistical differences in WBC, lymphocyte counts and MLR between all groups. Similar results were obtained by Celik et al., who revealed that both WBC, neutrophil, lymphocyte counts and NLR values were significantly higher in the group of patients with GO compared with the controls [28]. Contrary to the above-mentioned results, there are reports showing no significant differences in NLR values between GD patients as compared with healthy subjects [44]. Surprisingly, Dağdeviren et al. noted that neutrophil levels and NLR values were statistically lower in hyperthyroid patients with GD compared with non-GD hyperthyroid group and healthy individuals. Therefore, they stated NLR could not be a useful indicator for determining the etiology of hyperthyroidism. Lymphocyte counts were found to be higher in GD group than in the controls, but without statistical significance [45]. This finding is contrary to our study, as we noted significantly lower lymphocyte counts in GD patients compared to control group. However, the exact mechanisms responsible for the above-mentioned changes in blood counts have not been clarified yet. It is suggested that nearly all subpopulations of WBC as well as thrombocytes are engaged in the pathogenesis of that disease. T cells activation plays a pivotal role in the development of autoimmunity. T lymphocyte-dependent stimulation of B lymphocytes leads to the production of autoreactive antibodies which are responsible for the induction of fibroblast proliferation in the eye orbit, and the occurrence of GO symptoms. What is more, it was observed that activated T cells release cytokines, such as tumor necrosis factor α (TNF-α), interleukin 1 β (IL-1β), interleukin 6 (IL-6), and interleukin (IL-17), which results in the increased production and recruitment of neutrophils and macrophages. This hypothesis partially explains neutrophilia, increased monocyte counts, and MLR values in the course of GD which were observed in our research [5,12,28,29,44,46].

We displayed that PLR values were statistically higher in GD patients with or without GO than in healthy individuals. In contrast to obtained results, Dasgupta R et al. stated that PLR values were significantly statistically lower in GD patients than in the controls [26]. Similarly to Taskaldiran et al., we failed to find any distinctions in platelet counts between all GD patients and controls. Interestingly, our results confirmed that MPV values were statistically higher in all GD patients compared to controls, which was similarly reported by Taskaldiran et al. [27]. However, we noted no statistically significant intergroup differences in MPV values. MPV reflects not only platelet size, but also platelet activity, that is why its highest volume is observed in the GD patients with GO, while the lowest in healthy subjects. The pathomechanism of the above-mentioned disorders seems to be really confusing to explain. It is well-established that platelets actively react in the inflammatory processes. Some authors state that the level of platelet may be decreased in GD patients due to the immune-induced thrombocytopenia or hypersplenism in the course of GD [47]. Other researchers determined that the thrombocytopenia occurs as a result of shortening of platelet survival time with the simultaneous increase of megacariocyte proliferation in bone marrow [47].

In accordance to previous data, we proved that there were no statistically significant differences in basophil and eosinophil counts in GD patients with and without GO compared to controls [44,48]. Some reports suggested increased percentage of eosinophil in peripheral blood in GD patients compared to healthy individuals, but it might be associated with concomitant Th-2-predominant disorders [48,49,50].

The activity of GO may be assessed using some subjective scales, therefore more objective clinical tools and blood-derived biomarkers are needed. The current study revealed statistically significant higher WBC and neutrophil counts, and increased NLR, MLR values, decreased lymphocyte counts, but with no significance, in active GO compared to inactive GO. MPV/PLT was surprisingly statistically lower in active than in inactive GO, thus further studies are needed to evaluate usefulness of this ratio. This partially stands in agreement with Celik et al. who reported significantly higher WBC, neutrophil, lymphocyte counts, and NLR values in patients with active GO compared to inactive thyroid eye disease [28]. The similar profile of WBC system was described by Atilgan et al., although lymphocyte counts were statistically lower in patients with active GO [29]. It is considered that neutrophils are the active components of inflammatory process in autoimmune diseases and they are responsible for both initiation and maintenance of inappropriate immune responses as well as organ damage, whereas lymphocytes are treated as protective and inflammatory factors [51,52]. This confirms our findings that changes in peripheral blood cells might reflect increased inflammation in active GO. The potential inconsistence in analyzed parameters among mentioned studies may result from two different scales: CAS and VISA (V—vision, I—inflammation, S—strabismus, A—appearance), used to assess the activity and severity of GO [2]. The novelty of our study was the observation that the increase in GO activity reflected by the number of scores in the CAS scale is associated with the presence of higher WBC, neutrophil, monocyte counts, and NLR values, which confirms the usefulness of that non-invasive and cheap markers in the assessment of the course of GO. The symptoms which were most commonly reported by our patients, such as redness of the conjunctiva and ocular pain on attempted upward or downward gaze, were associated with the higher level of WBC, neutrophil, and NLR as well as higher NLR and PLR values, respectively. No literature data regarding correlation between hematological parameters and particular symptoms in CAS scale was found, therefore further research is necessary.

According to our knowledge, we assessed the impact of smoking on hematological parameters in GD patients with and without GO for the first time. The study revealed that current smokers with GO displayed the highest WBC and neutrophil counts compared with former smokers and never smokers. Taking into consideration simultaneously three smoking statuses and the division of the whole population into particular groups (GD with/without GO, the controls), WBC and neutrophil counts were also the highest in active smokers with GO. Due to the fact that the effect of smoking as a risk factor for GD and GO development is stronger in current smokers than in former smokers, and due to the proven reversible changes in CBC-derived parameters after cessation of tobacco use, we decided to include former smokers and never smokers to one non-smoker group [3,34]. Smokers with GO displayed statistically higher WBC, neutrophil counts and lower lymphocyte counts compared to patients with GD without GO and controls. Moreover, smokers with GD without GO showed higher PLR values, similarly to smokers in the control group compared to non-smokers. The inflammatory effects of smoking on CBC have been proven in previous studies using different biomarkers, although its impact on NLR, MLR, PLR, MPV/PLT values is still not fully understood, and available researches were conducted mainly among healthy population. Tulgar et al., Gumus et al., and Çekici et al. demonstrated statistically significant higher WBC, neutrophil, eosinophil counts, and NLR values in smokers than in non-smokers [53,54,55]. In most cases monocyte counts were increased, whereas lymphocyte counts were decreased with no significant differences in smokers, and only Gumus et al. showed significantly higher lymphocyte counts in smoking patients, similarly to our results [54]. There was no clear consensus on PLT counts, MPV, MPV/PLT, and PLR values among smokers and non-smokers in the above studies. Higher WBC and lymphocyte counts, which were observed in GD patients, may suggest active smoking and indicate the risk of a severe course of the disease or greater likelihood of developing GO. Moreover, positive linear correlation was found between the pack-years, reflecting the number of cigarettes smoked daily and the time of smoking, and NLR [53,55]. However, our study revealed the negative linear correlation with the number of cigarettes/day as well as duration of tobacco use and platelet and PLR values in GO group, which may be caused by nicotine-induced decreased thrombopoietic activity in smokers [56]. Smokers with GD who present higher values of WBC, neutrophil, eosinophil counts and NLR values can be easily identified during routine morphology check-up, and may benefit from preventive actions. Notwithstanding, the mechanisms by which smoking stimulates inflammatory processes and reflects their intensity by changes in CBC-derived hematological parameters are largely unknown so far. It is considered that hypoxia occurring during active tobacco use, and stimulating fibroblasts proliferation, glycosaminoglycan production, and differentiation into adipocytes may be partially responsible for the link between smoking and GO development [10]. The studies displayed that smokers were characterized by increased release of leukocytes and platelets into peripheral blood which was a consequence of stimulation of hematopoietic system in bone marrow, and in this way it exaggerated systemic and local inflammatory response [10]. Furthermore, exposure to cigarette smoke affected both the innate and adaptive immune systems, and in consequence favoured the occurring of GO. The studies revealed that the activation of neutrophils, macrophages, and epithelial cells as well as releasing of pro-inflammatory cytokines, inhibiting anti-inflammatory mediators, reactive oxygen species (ROS) resulted in increased oxidative stress, cells and tissues damages [3,10]. Therefore, the search for other underlying mechanistic pathways behind above theories in the course of GD with and without GO should be the subject of further research.

In the present study we revealed that NLR values and WBC counts may be factors increasing the risk of GO in GD patients with OR 1.348 and OR 1.209, respectively, and that is why these hematological parameters can be additional tools to determine the risk of GO occurrence, beside assessing smoking habits. Moreover, we would like to highlight that the differences in hematological parameters, observed in our retrospective study, are interesting epidemiologically and for hypothesis generating, although they are unspecific to be currently applied at the clinical practice.

## 5. Limitations of the Study

This cross-sectional study has some limitations, mainly due to the retrospective nature of the research. Firstly, the study was not designed to discover the pathways that lead to the changes in NLR, MLR, PLR, MPV/PLT or MPV values as we had no data about further well-established inflammatory markers to compare our results, such as highly sensitive CRP (hs-CRP), erythrocyte sedimentation rate (ESR), TNF-α, IL-1β, IL-6. Secondly, the routine measurement of MPV has been introduced in our laboratory since 2011, that is why MPV/PLT ratio was not evaluated in all patients. Another limitation was the inability to assess all GD patients at the time of diagnosis and after long-term follow-up, which could provide an accurate information about correlations between changes in hematological parameters and TRAb concentrations.

## 6. Conclusions

The results of the current study indicate that CBC-derived inflammatory markers differ significantly in GD patients depending on the presence of GO, and as compared with healthy subjects. The detection of high NLR, MLR, and PLR values during routine laboratory testing might help in early prediction of the inflammation process development as well as stress and tissue damage. NLR values and WBC counts may help select the group of patients with the highest risk of GO development in the course of GD, which entail increased endocrine and radiological controls. The increase in WBC counts, neutrophilia, and lymphocytopenia, reflected by high NLR values, is significantly associated with GO activity.

WBC, neutrophil, lymphocyte counts, PLR and MPV values may also reflect worsening of the existing inflammatory and autoimmune condition by smoking in GD patients with and without GO, thus monitoring the levels of these indices may help motivate efforts to stop tobacco use. Therefore, evaluation of NLR, MLR, and PLR as novel, non-invasive, and widely accessible markers in the context of diagnostic approach, clinical assessment and management of GO should remain active areas of research.

## Figures and Tables

**Table 1:** General characteristics of the study and control groups.

Variables	GD with GO	GD without GO	Controls	*p*-Value
	(*n* = 168)	(*n* = 238)	(*n* = 100)	
Age [years]	50.4 ± 12.9	46.7 ± 16.5	42.5 ± 17.3	<0.001 * ^1^
Female, *n* (%)	138 (82)	193 (81)	65 (65)	0.002 * ^2^
Male, *n* (%)	30 (18)	45 (19)	35 (35)	
BMI [kg/m^2^]	26.1 ± 5.4	25.3 ± 4.8	25.5 ± 4.1	0.280 ^1^
Duration of GD [months]	52.3 ± 74.4	34.9 ± 66.3	-	0.013 * ^3^
Duration of GO [months]	20.7 ± 47.6	-	-	-

Values are number (%) or mean ± SD. * *p*-value < 0.05; ^1^ one-way ANOVA; test ^2^ Chi-Squared test; ^3^ Student’s *T*-test.

**Table 2:** Baseline characteristics of laboratory data among three groups.

Variables	Group 1 GD with GO	Group 2 GD without GO	Group 3 Controls	*p*-Value	p (Groups 1&2)	p (Groups 1&3)	p (Groups 2&3)
	(*n* = 168)	(*n* = 238)	(*n* = 100)				
WBC [10^9^/L]	6.64 ± 2.09	5.96 ± 1.56	6.10 ± 0.90	0.014 *	0.022 *	NS	NS
NEU [10^9^/L]	3.78 ± 1.42	3.24 ± 1.06	3.11 ± 0.46	<0.001 *	<0.001 *	<0.001 *	NS
LYM [10^9^/L]	2.07 ± 0.79	1.94 ± 0.64	2.23 ± 0.43	0.008 *	NS	NS	0.005 *
NLR	1.95 ± 0.77	1.76 ± 0.62	1.41 ± 0.21	<0.001 *	0.018 *	<0.001 *	<0.001 *
EOS [10^9^/L]	0.17 ± 0.12	0.18 ± 0.20	0.18 ± 0.11	0.663	NS	NS	NS
BASO [10^9^/L]	0.03 ± 0.02	0.03 ± 0.05	0.03 ± 0.02	0.881	NS	NS	NS
MONO [10^9^/L]	0.43 ± 0.31	0.46 ± 0.28	0.41 ± 0.13	0.156	NS	NS	NS
MLR	0.22 ± 0.14	0.25 ± 0.23	0.19 ± 0.06	0.010 *	NS	NS	0.033 *
PLT [10^9^/L]	252.88 ± 65.07	257.57 ± 70.42	248.57 ± 64.36	0.997	NS	NS	NS
PLR	135.63 ± 53.04	141.77 ± 48.05	113.64 ± 31.56	<0.001 *	NS	0.003 *	<0.001 *
MPV [fL]	8.21 ± 1.13	8.11 ± 1.04	7.87 ± 0.89	0.054	NS	NS	NS
MPV/PLT	0.04 ± 0.01	0.04 ± 0.01	0.03 ± 0.01	0.567	NS	NS	NS

MPV: GD with GO *n* = 107, GD without GO *n* = 141, controls *n* = 100; * *p*-value < 0.05 was statistically significant. One-way ANOVA with Tukey’s post hoc test for unequal *n* was used in the analysis. NS—no significance; GD—Graves’ disease; GO—Graves’ orbitopathy; WBC—white blood cell; NEU—neutrophil; LYM—lymphocyte; NLR—neutrophil-to-lymphocyte ratio; EOS—eosinophil; BASO—basophil; MONO—monocyte; MLR—monocyte-to-lymphocyte ratio; PLT—platelet; PLR—platelet-to-lymphocyte ratio; MPV—mean platelet volume.

**Table 3:** Analysis of selected CBC parameters and ratios in GO patients according to particular signs from CAS scale and subjective symptoms of GO.

CBC Parameters and Ratios	CAS Scale							Subjective Symptoms of GO		
	Spontaneous Retrobulbar Pain	Ocular Pain on Attempted Upward or Downward Gaze	Redness of the Eyelids	Redness of the Conjunctiva	Swelling of the Eyelids	Chemosis of the Conjunctiva	Inflammation of Caruncule and/or Plica **	Excessive Tearing or Dry Eyes	Diplopia	Photophobia
WBC [10^9^/L]	6.80 ± 2.14 0.295	6.19 ± 1.87 0.050 *	6.82 ± 2.09 0.531	6.84 ± 2.17 0.012 *	6.66 ± 2.10 0.930	6.70 ± 2.11 0.483	**	6.66 ± 2.13 0.890	7.00 ± 2.07 0.022 *	7.14 ± 1.50 0.222
NEU [10^9^/L]	3.93 ± 1.45 0.115	3.60 ± 1.33 0.260	4.02 ± 1.53 0.188	3.92 ± 1.46 0.005 *	3.80 ± 1.38 0.804	3.81 ± 1.48 0.568	**	3.81 ± 1.41 0.636	3.94 ± 1.33 0.131	4.21 ± 1.08 0.115
LYM [10^9^/L]	2.08 ± 0.79 0.811	1.85 ± 0.68 0.013 *	1.99 ± 0.86 0.490	2.10 ± 0.83 0.220	2.06 ± 0.83 0.886	2.07 ± 0.77 0.860	**	2.04 ± 0.83 0.484	2.21 ± 0.80 0.017 *	2.13 ± 0.62 0.670
NLR	2.03 ± 0.83 0.144	2.12 ± 0.99 0.050 *	2.17 ± 0.84 0.031 *	2.00 ± 0.80 0.050 *	1.99 ± 0.83 0.400	1.96 ± 0.81 0.769	**	2.00 ± 0.80 0.119	1.93 ± 0.84 0.713	2.13 ± 0.88 0.224
EOS [10^9^/L]	0.18 ± 0.11 0.195	0.14 ± 0.11 0.029 *	0.16 ± 0.10 0.553	0.16 ± 0.11 0.012 *	0.17 ± 0.11 0.956	0.17 ± 0.11 0.391	**	0.16 ± 0.11 0.257	0.18 ± 0.12 0.234	0.17 ± 0.12 0.970
BASO [10^9^/L]	0.03 ± 0.02 0.950	0.03 ± 0.02 0.636	0.03 ± 0.02 0.978	0.03 ± 0.02 0.021 *	0.03 ± 0.02 0.228	0.03 ± 0.02 0.151	**	0.03 ± 0.02 0.466	0.04 ± 0.02 0.043 *	0.03 ± 0.01 0.965
MONO [10^9^/L]	0.47 ± 0.39 0.102	0.40 ± 0.20 0.403	0.50 ± 0.55 0.067	0.45 ± 0.34 0.144	0.45 ± 0.39 0.373	0.44 ± 0.33 0.500	**	0.44 ± 0.35 0.334	0.47 ± 0.40 0.060	0.46 ± 0.26 0.619
MLR	0.24 ± 0.18 0.088	0.23 ± 0.11 0.488	0.27 ± 0.24 0.009 *	0.23 ± 0.15 0.346	0.23 ± 0.17 0.371	0.23 ± 0.16 0.394	**	0.23 ± 0.16 0.132	0.23 ± 0.18 0.702	0.23 ± 0.15 0.646
PLT [10^9^/L]	254.23 ± 65.56 0.770	246.60 ± 68.41 0.385	254.79 ± 70.27 0.824	252.17 ± 67.13 0.768	256.75 ± 68.64 0.391	250.26 ± 65.47 0.337	**	247.29 ± 67.74 0.058	256.94 ± 62.26 0.408	263.39 ± 64.50 0.406
PLR	135.05 ± 52.49 0.878	148.66 ± 63.84 0.026 *	142.21 ± 55.31 0.347	133.84 ± 54.25 0.360	138.55 ± 55.02 0.429	134.26 ± 54.91 0.538	**	135.03 ± 54.33 0.801	129.43 ± 53.63 0.121	133.95 ± 51.02 0.871
MPV [fL]	8.29 ± 1.10 0.351	8.02 ± 1.06 0.220	8.03 ± 1.10 0.278	8.21 ± 1.16 0.984	8.01 ± 1.02 0.021 *	8.36 ± 1.19 0.035 *	**	8.22 ± 1.19 0.826	8.14 ± 1.01 0.450	7.93 ± 0.97 0.269
MPV/PLT	0.04 ± 0.01 0.853	0.04 ± 0.01 0.748	0.03 ± 0.01 0.246	0.04 ± 0.01 0.928	0.03 ± 0.01 0.179	0.04 ± 0.01 0.846	**	0.04 ± 0.01 0.601	0.04 ± 0.01 0.568	0.03 ± 0.01 0.389

Data are presented as mean ± SD (*p*-value). MPV: GD with GO *n* = 107. * *p*-value < 0.05 was statistically significant. ** due to single observation of this sign in the studied group, the statistical analysis is impossible. Student’s *T*-test was applied. GO—Graves’ orbitopathy; WBC—white blood cell; NEU—neutrophil; LYM—lymphocyte; NLR—neutrophil-to-lymphocyte ratio; EOS—eosinophil; BASO—basophil; MONO—monocyte; MLR—monocyte-to-lymphocyte ratio; PLT—platelet; PLR—platelet-to-lymphocyte ratio; MPV—mean platelet volume.

**Table 4:** Univariate and multivariate linear regression analysis of the association between hematological inflammatory markers and sociodemographic parameters and the risk of GO.

Variable	Univariate		Multivariate	
	OR (95% CI)	*p*-Value	OR (95% CI)	*p*-Value
Female	1.059 (0.635–1.766)	0.825		
Smoking	1.656 (1.084–2.531)	0.020 *		
WBC	1.232 (1.101–1.380)	<0.001 *	1.209 (1.078–1.355)	0.001 *
NEU	1.421 (1.203–1.680)	<0.001 *		
LYM	1.280 (0.967–1.693)	0.084		
NLR	1.468 (1.093–1.970)	0.011 *	1.348 (1.078–1.355)	0.048 *
EOS	0.613 (0.167–2.243)	0.459		
BASO	9.797 (0.046–2081.645)	0.404		
MONO	0.720 (0.336–1.542)	0.398		
MLR	0.285 (0.052–1.579)	0.151		
PLT	0.999 (0.996–1.002)	0.531		
PLR	0.998 (0.994–1.002)	0.239		
MPV	1.096 (0.868–1.385)	0.441		
MPV/PLT	0.021 (0.000–6175859.283)	0.698		

* *p*-value < 0.05 was statistically significant. OR—odds ratio; CI—confidence Interval; WBC—white blood cell; NEU—neutrophil; LYM—lymphocyte; NLR—neutrophil-to-lymphocyte ratio; EOS—eosinophil; BASO—basophil; MONO—monocyte; MLR—monocyte-to-lymphocyte ratio; PLT—platelet; PLR—platelet-to-lymphocyte ratio; MPV—mean platelet volume.

## Supplementary Materials

The following are available online at https://www.mdpi.com/2077-0383/9/10/3111/s1, Table S1: Comparison of hematological data between all GD patients and controls, Figure S1A–L: Comparison of hematological parameters and ratios between active (A) and inactive (N) GO groups.

## Abbreviations

GD	Graves’ disease
GO	Graves’ orbitopathy
CAS	Clinical Activity Score
CBC	complete blood count
WBC	white blood cell
NEU	neutrophil
LYM	lymphocyte
EOS	eosinophil
BASO	basophil
MONO	monocyte
NLR	neutrophil-to-lymphocyte ratio
MLR	monocyte-to-neutrophil ratio
PLR	platelet-to-lymphocyte ratio
MPV	mean platelet value
MPV/PLT	mean platelet volume-to-platelet ratio
BMI	Body Mass Index
TRAb	anti-TSH receptor antibody
TSH	thyroid stimulating hormone
fT3	free triodothyronine
fT4	free thyroxine
