# Supplementary material for: Neutrophil-to-Lymphocyte, Monocyte-to-Lymphocyte and Platelet-to-Lymphocyte Ratios in Relation to Clinical Parameters and Smoking Status in Patients with Graves’ Orbitopathy—Novel Insight into Old Tests
Source: J Clin Med. 2020 Sep 26;9(10):3111. doi: 10.3390/jcm9103111 (PMC7600876; doi:10.3390/jcm9103111)
Supplement: Supplementary file 1 [file jcm-09-03111-s001.pdf]

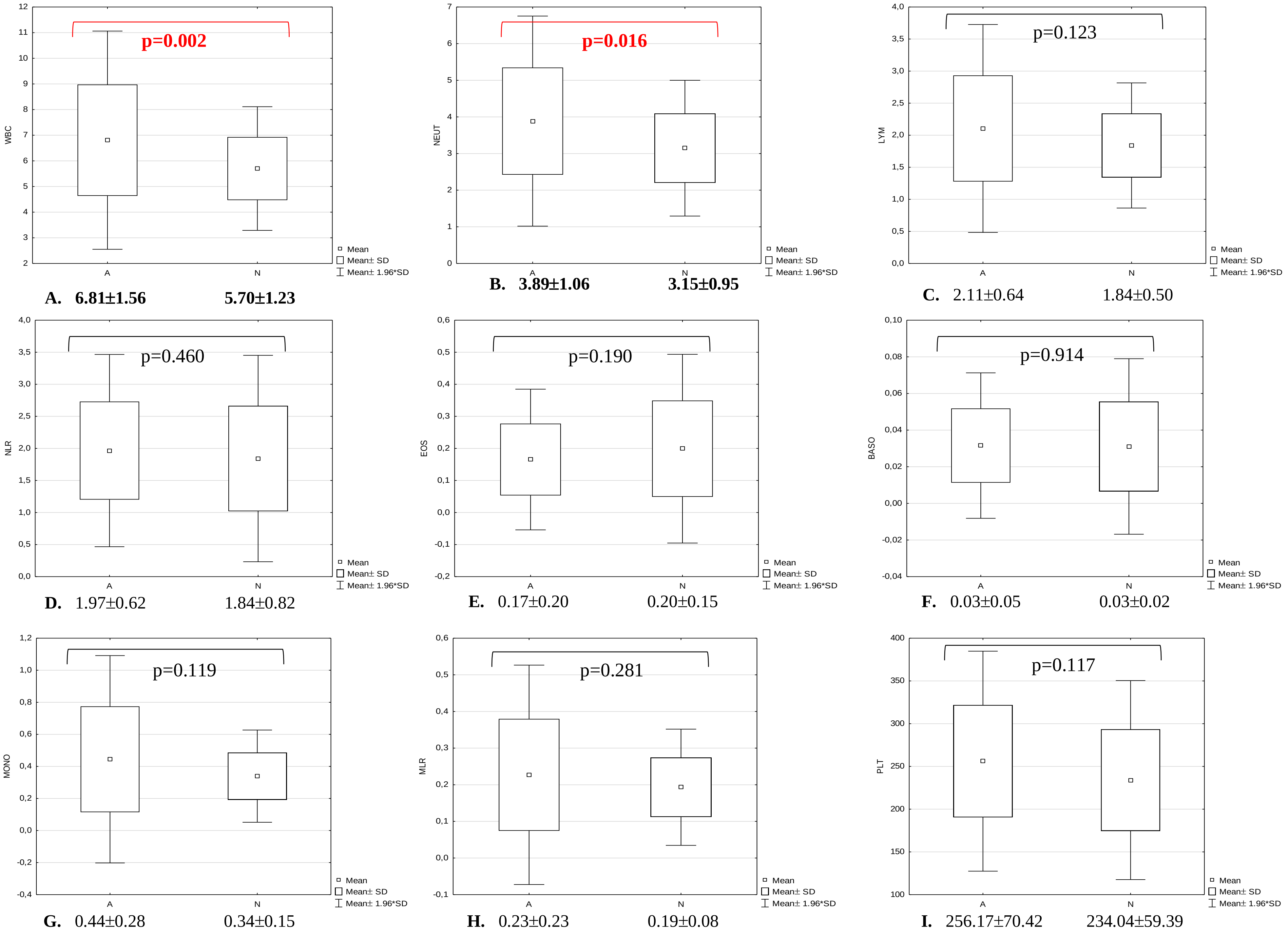

WBC
p=0.002
A
N
Mean
Mean± SD
Mean± 1.96*SD
A. 6.81±1.56
5.70±1.23
NEUT
p=0.016
B. 3.89±1.06
3.15±0.95
LYM
p=0.123
C. 2.11±0.64
1.84±0.50
NLR
p=0.460
D. 1.97±0.62
1.84±0.82
EOS
p=0.190
E. 0.17±0.20
0.20±0.15
BASO
p=0.914
F. 0.03±0.05
0.03±0.02
MONO
p=0.119
G. 0.44±0.28
0.34±0.15
MLR
p=0.281
H. 0.23±0.23
0.19±0.08
PLT
p=0.117
I. 256.17±70.42
234.04±59.39

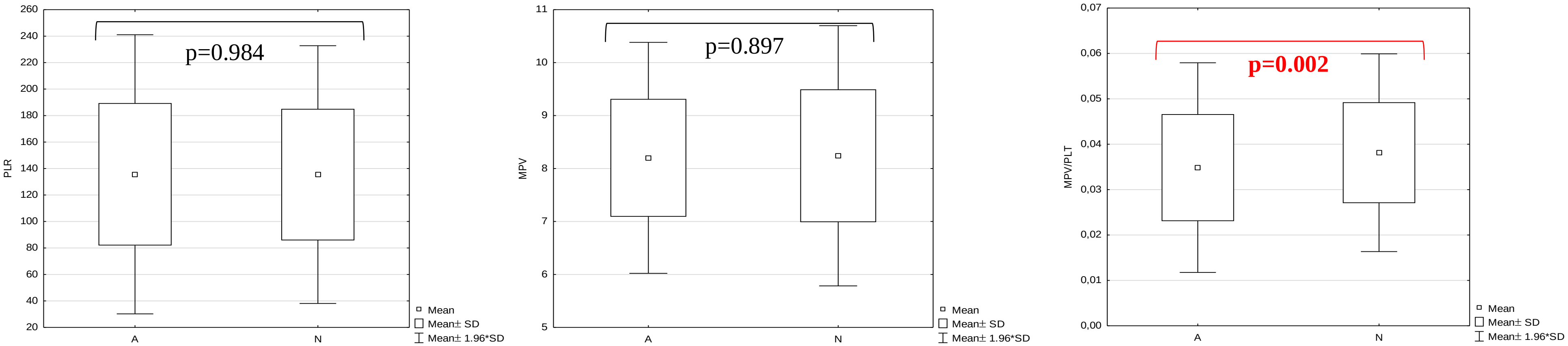

J. 135.67±48.05 135.43±49.67 **K.** 8.20±1.04 8.24±1.25 **L.** **0.03±0.01** **0.04±0.01**

Figure S1. Comparison of hematological parameters and ratios between active (A) and inactive (N) GO groups.
(**A-B,L**). There was the statistically significant differences in WBC, NEU counts and MPV/PLT values in patients with active and inactive GO (6.81 ± 1.56 vs 5.70 ± 1.23, p=0.002; 3.89 ± 1.06 vs 3.15 ± 0.95, p=0.016; 0.03 ± 0.01 vs 0.04 ±0.01, p=0.002). (**C-K**). No statistically significances were observed in cases of LYM, NLR, EOS, BASO, MONO, MLR, PLT, PLR, MPV. p-value <0.05 was statistically significant.
GO – Graves' orbitopathy; WBC – white blood cell; NEU – neutrophil; LYM – lymphocyte; NLR – neutrophil-to-lymphocyte ratio; EOS – eosinophil; BASO – basophil; MONO – monocyte; MLR – monocyte-to-lymphocyte ratio; PLT – platelet; PLR – platelet-to-lymphocyte ratio; MPV – mean platelet volume

Table S1. Comparison of hematological data between all GD patients and controls.

Variables	GD patients		Controls		p-value
	Mean	SD	Mean	SD	
WBC [10^9/L]	6.24	1.83	6.10	0.90	<0.001 *
NEUT [10^9/L]	3.46	1.25	3.11	0.46	<0.001 *
LYM [10^9/L]	1.99	0.71	2.23	0.43	<0.001 *
NLR	1.84	0.69	1.69	1.21	0.095
EOS [10^9/L]	0.18	0.17	0.18	0.11	0.866
BASO [10^9/L]	0.03	0.04	0.03	0.02	0.236
MONO [10^9/L]	0.44	0.29	0.41	0.13	0.317
MLR	0.24	0.20	0.18	0.07	0.004 *
PLT [10^9/L]	255.63	68.22	248.57	64.36	0.349
PLR	139.23	50.20	107.70	39.88	<0.001 *
MPV [fL]	8.15	1.08	7.87	0.89	0.028 *
MPV/PLT	0.04	0.01	0.03	0.01	0.322

* p-value <0.05 was statistically significant. Student's T-test was applied.

GD – Graves' disease; WBC – white blood cell; NEU – neutrophil; LYM – lymphocyte; NLR – neutrophil-to-lymphocyte ratio; EOS – eosinophil; BASO – basophil; MONO – monocyte; MLR – monocyte-to-lymphocyte ratio; PLT – platelet; PLR – platelet-to-lymphocyte ratio; MPV – mean platelet volume
